# Digital Dance for People With Parkinson's Disease During the COVID-19 Pandemic: A Feasibility Study

**DOI:** 10.3389/fneur.2021.743432

**Published:** 2022-02-03

**Authors:** Lois Walton, Magdalena Eriksson Domellöf, Åsa N. Åström, Åsa Elowson, Anna Stigsdotter Neely

**Affiliations:** ^1^Department of Social and Psychological Studies, Karlstad University, Karlstad, Sweden; ^2^Department of Psychology, Umeå University, Umeå, Sweden; ^3^Balettakademien Stockholm, Stockholm, Sweden; ^4^Kognitiva Teamet Rehab, Stockholm, Sweden; ^5^Engineering Psychology, Luleå University of Technology, Luleå, Sweden

**Keywords:** Parkinson's disease, telemedicine, dance & movement, quality of life, mixed method analysis, feasibility studies

## Abstract

**Background:**

Dance as a treatment to support physical, cognitive and emotional functioning, has gained increased acceptance as a healthcare intervention for people with Parkinson's Disease (PD). The impact of the COVID-19 pandemic has been far reaching with devastating effects for at-risk populations. To find alternative and safe treatment delivery options during the pandemic has been of utmost importance. The purpose of this mixed-methods study was to evaluate the feasibility and the experience of digital dance for people with PD (Dance for PD©) and to examine change in self-reported quality of life, psychological health, subjective cognitive complaints and mental fatigue.

**Methods:**

23 participants with PD (mean age 70) partook in 10-h weekly digital Dance for PD sessions. Feasibility outcome measures were assessed at post-test. Web-based questionnaires examining quality of life, subjective memory complaints, depression, anxiety and mental fatigue were administered at pre- and post-test. Moreover, nine participants partook in focus group discussions at post-test.

**Results:**

The results showed an acceptable feasibility to home-based digital Dance for PD, where 86% of the dance classes were completed, only minor negative side effects were reported (i.e., sore joints), and all experienced the dance classes as motivating and safe to do at home. The majority also reported positive effects on mood and physical functioning. The results from the questionnaires showed significant improvements in depressive symptoms (*p* = 0.006) and quality of life (*p* < 0.001) at post-test. In the focus groups, participants indicated that digital dance was a beneficial and enjoyable activity with a strong added value during the COVID-19 pandemic. Nevertheless, they experienced that digital dance missed some important elements of live dance.

**Conclusions:**

This study showed that digital Dance for PD is feasible and holds promise as a viable and safe method to keep people with PD dancing even when physical meetings are not possible. Beyond the pandemic, digital dance could be applied to a wide variety of patient groups including rural populations and patients for whom transportation may not be feasible for practical or financial reasons.

**Trial Registration:**

Retrospectively registered at ClinicalTrials.gov on 25/06/2021 with the following registration number: NCT04942392.

## Introduction

Parkinson's Disease (PD) affects 6.1 million people worldwide, which is more than a two-fold increase since 1990 (1). A variety of symptoms occur throughout the disease course with diagnosis being based on motor symptoms, i.e., bradykinesia, rigidity and tremor (2). However, both motor and non-motor symptoms, such as cognitive decline and depression, negatively affect quality of life (3). Pharmacological treatment in the form of Dopamine Replacement Therapy (DRT) aims to increase dopaminergic availability and shows positive effects on motor symptoms, yet its influence on non-motor symptoms is variable (4). Furthermore, the effect of DRT decreases over time, leading to higher medication dosages and more side effects (5).

Non-pharmacological interventions have been stated as essential during the early phase of PD (6) and an important complement to DRT in later stages of the disease (7). Dance is especially interesting due to its combination of movement, cognition and rhythmic stimulation (8), thereby engaging both motor and non-motor elements of PD. While several recent meta-analyses on the effects of dance for people with PD have reported improved postural control, functional mobility and overall motor symptoms (9–14), dance's effect on non-motor symptoms and quality of life has received less attention and is currently undetermined (9, 10, 12, 15, 16). Future studies have specifically been advocated to incorporate a mixed-methods design in order to better understand the impact of dance for people with PD in their daily lives (10, 13).

Beyond the cognitive and physical element to dance, people with PD have reported changes in body experience and a sense of connection to others during dance (17, 18). Moreover, both positive and negative emotions related to dance have been described where feelings of enjoyment and an uplifted mood as well as feelings of frustration and embarrassment in relation to physical disabilities have been reported (18, 19). Understanding the meaning of dance for people with PD is important, both from a patient-centered perspective and in order to better understand motivation and compliance regarding dance interventions.

Even though evidence for the benefits of exercise in PD is strong, people with PD and the older population in general report that they experience barriers to participate, such as fear of falling, lack of motivation or transport difficulties (20, 21). The COVID-19 pandemic amplified and added to these barriers, increasing the risk of social isolation and consequentially its negative effect on overall well-being (22). Providing social and joyful exercise-based interventions through a digital platform at home could therefore be an alternative for people with PD. Recent studies on tele-rehabilitation and home-based exercise programs have shown that such digital solutions are safe and may display similar health-related benefits compared to face-to-face non-pharmacological interventions (23, 24).

The goal of this mixed-methods study was three-fold. The study aimed to examine: (1) the feasibility and safety of a digital home-based dance intervention for people with PD; (2) change in self-reported quality of life, cognitive complaints, psychological health and fatigue; and (3) the experience of digital dance for people with PD.

## Method

### Design

The study has a one-group pre- and post-test design.

### Participants

This study included 23 participants recruited from the Dance for Parkinson's Disease classes (DfPD©) held at Balettakademien in Stockholm (Sweden) during September—December 2020. The dance classes were subsidized by Region Stockholm and the registration fee was 400 SEK. Inclusion criteria were self-reported diagnosis of PD and being registered as a participant of Balettakademien's DfPD dance class.

Thirty seven people with PD signed up for the dance class and received an information letter *via* email describing the purpose of the research project and an invitation to participate. Those who agreed to participate (*n* = 33) received a link to a web questionnaire before the first dance class of the 33 participants answering the digital questionnaire at pre-test, 23 answered the questionnaires at post-test. These 23 participants had a mean age of 70.4, 74% were women, mean years with PD was 8.3 years, 57% reported being in good to very good health and 57% had previous experience of DfPD (see Table 1). Drop-out analyses were conducted and there were no significant differences between the 23 participants who answered both the pre- and post-test questionnaires compared to those who only answered the pre-test questionnaires in regard to age, years of education, years with PD, gender, previous dance experience and general health (*ps* > 0.300 for all the former), as well as depression (*p* > 0.200). The reasons for not participating at post-test are unknown, however some participants experienced problems with the link to the web questionnaire.

**Table 1:** Background variables for the 23 participants who answered the questionnaire at both pre- and post-test.

**Background**	**Mean (SD) or *N* (%)**	**Min-Max**
Age	70.4 (7.4)	55–85
Years with PD	8.0 (6.4)	1–26
Sex (f/m)	17/6 (74%)	
High school	22 (96%)	
University	19 (83%)	
Self-evaluated health (good or very good)	13 (57%)	
Previous experience of Dance for PD	13 (57%)	
Other physical activity	10 (44%)	

### Digital Dance Class

The digital dance class was held *via* Zoom and taught by a professional, certified DfPD and experienced dance instructor. The dance classes followed the DfPD protocol but were adjusted to fit the digital format. The Zoom room was opened 30 mins before class for social interaction and information. The classes were scheduled once a week for 60 mins and consisted of 10 sessions. The total number of participants per digital dance class ranged from 15 to 20.

Each dance class started with a 5 min warm-up without music, focusing on awareness of one's own body and the room. This was followed by 20–25 mins of seated dance techniques including upper and lower body movement with improvisational and choreographed sequences requiring memorization. After a 2-min water break, 20–25 mins of standing movements were performed, supported by holding the back of a chair (or seated if preferred), consisting of Ballet, Flamenco and Contemporary dance exercises, theatrical interpretation, improvisational as well as choreographed sequences requiring memorization. Rhythmic patterns were exercised throughout the class. All parts of the dance class could be adapted to the participant's physical and mental state, e.g., performing movements while seated. The dance class ended with 5 mins of winding down exercises, again focusing on one's own body and acknowledging the other dancers and the shared experience. The Zoom room was open for 10–15 mins after class for questions and reflections.

To ensure a safe digital dance experience, the participants were requested at the beginning of each class to have a sturdy chair available for support and a phone nearby if the participant was alone in their home during dancing. Furthermore, the dance instructor and participants agreed to have the video function running during the whole dance class. In the case of a participant disappearing from view, a telephone number to a designated contact person was available to the dance instructor. Participants were reminded repeatedly before and during the dance class that the main focus is that they feel safe and enjoy themselves. Performing the dance steps while seated was provided as an alternative in all exercises.

### Outcomes Measures

#### Feasibility Measures

Based on the guidelines by Bowen et al. (25) and inspired by Van De Weijer et al. (26), the following feasibility outcome measures were assessed at post-test: (a) technical and safety issues, (b) compliance and motivation, (c) self-assessed improvement in physical function, well-being, and cognitive function, and (d) the importance of dance as an artistic and creative activity. In order to judge the success rate of each feasibility outcome measure, a criterion of success was set. An overview of the feasibility measures can be found in Table 2. The questions were rated on 5-point Likert scales raging from (1 = very unsatisfactory, 5 = very satisfactory), (1 = not motivated, 5 = very motivated), (1 = noticeable worsening, 5 = noticeable improvement) or (1 = not meaningful, 5 = very meaningful), as well as the opportunity for open-ended answers. Two open-ended questions regarding positive and negative aspects of digital dancing were also included.

**Table 2:** Overview of the feasibility outcome measures assessed at post-test.

**Feasibility measures**	**Questions**	**Data collected**	**Feasibility criterion for success**	**Results**	**Success**
Accessibility/technical smoothness	In general, how has dancing *via* Zoom worked?	5-point Likert scale: 1 (very unsatisfactory) - 5 (very satisfactory)	70% OK to very satisfactory	70% reported satisfactory to very satisfactory. 26% responded with OK. One participant reported unsatisfactory. Negative reports were based on technical issues such as unsynchronized sound, feeling distance to the group, limited screen size. Median = 5.0	Yes
Safety	Has digital dance been perceived as safe to perform at home?	Yes or No	100% Yes	All participants considered digital dance to be safe.	Yes
Side effects	Has digital dance caused negative side effects?	Yes or No	100% reporting no side effects	90% reported no side effects. The reported side effects were sore joints which can be expected after physical exercise.	Yes
Training compliance	Number of attended dance classes	Number of attended dance classes (range 0–10)	60%	Mean percentage of the attended dance classes for the 23 participants at post-test was 86%.	Yes
Training motivation	How motivated have you been to participate in the dance classes?	5-point Likert scale: 1 (not motivated)−5 (very motivated)	80% sufficiently to very motivated	83% reported being sufficiently motivated to very motivated to attend dance class. Median = 5.0	Yes
Improved physical function	Do you feel that digital dance has affected your physical function?	5-point Likert scale: 1 (clear worsening)−5 (clear improvement)	No report of worse physical function	74% reported improved physical function and 26% reported unchanged physical function. Median = 4.0	Yes
Improved well-being	Do you feel that digital dance has affected your overall well-being?	5-point Likert scale: 1 (clear worsening)−5 (clear improvement)	No report of worse well-being	78% reported improved overall well-being and 22% reported unchanged overall well-being. Median = 4.0	Yes
Improved cognitive function	Do you feel that digital dance has affected your memory and attention?	5-point Likert scale: 1 (clear worsening)−5 (clear improvement)	No report of worse cognitive function	30% reported improved memory and attention 70% reported unchanged memory and attention Median = 3.0	Yes
The importance of dance as an artistic and creative activity	How important has the artistic expression been for your experience of digital dance?	5-point Likert scale: 1 (not meaningful)-−5 (very meaningful)	Not applicable	74% reported the artistic and creative dimension as meaningful. One person reported the artistic dimension as not at all meaningful. Median = 5.0	Not applicable

#### Cognitive Complaints, Fatigue, Psychological Health and Quality of Life

The following questionnaires were completed at pre- and post-test *via* an online survey tool:

The Hospital Anxiety Depression scale (HADS) was used to assess symptoms of anxiety and depression (27). This questionnaire consists of seven items about anxiety (Cronbach's alpha: 0.829) and seven items about depression (Cronbach's alpha: 0.789) that are scored on a 4-point Likert scale (0–3), total score (range 0–21).

The Prospective Retrospective Memory Questionnaire (PRMQ) was used to assess subjective cognitive complaints (28). This questionnaire consists of 16 items on memory slips in everyday life, i.e., eight on prospective memory failures (e.g., “Do you decide to do something in a few minutes time and then forget to do it?”) and eight on retrospective memory failures (e.g., “Do you fail to recognize a place you have visited before?”). Answers were given on a 5-point Likert scale with 1 = never and 5 = very often, total score (range 16–80) (Cronbach's alpha: 0.938).

Two questions from the Mental Fatigue Scale (29) were used to measure mental fatigue (item 3) and recovery (item 4). Answers were given on a 7-point Likert scale.

The Checklist Individual Strength (CIS) was used to measure level of fatigue (30). The questionnaire consists of 20 items rated on a 7-point Likert scale (1 = “yes, that is true”, 7 = “no, that is not true”). A total score was calculated (range 20–140), with a higher score indicating more fatigue (Cronbach's alpha: 0.887).

The Parkinson's Disease Questionnaire (PDQ-39) was used to assess function and well-being related to PD (31). This questionnaire consists of 39 items covering the following areas: mobility, activities of daily living, emotional well-being, stigma, social support, cognition, communication and bodily discomfort. The participant indicates the frequency of a certain event by answering on a 5-point Likert scale (range 0–4), total score (range 0–156) and score per subscale (Cronbach's alpha of total score: 0.936).

#### Focus Group Interviews

During the final dance class, participants were invited to partake in a focus group interview over Zoom. Two focus groups led by ASN and MD were held during 1 h, addressing the experience of digital dance. Each focus group consisted of 4–5 participants.

### Data Analysis

#### Quantitative Data Analysis

The quantitative data (pre-post questionnaires) was analyzed using IBM SPSS version 26. Paired sample *t*-tests were used to measure the difference between pre- and post-test for all outcome measures except the two items from the Mental Fatigue Scale for which the Wilcoxon Signed Rank *t*-test was used. Cohens D were calculated to assess the effect size of the change according to the following formula: D = [(M_pre−_M_post_) / (SD)] with SD = *SD*_*pre*_ $\sqrt{2\;\left(1-r\right)}$ and *r* being the correlation between the pre- and post-test (32). If the total score for a questionnaire was significantly different at post-test, an analysis of the score on subscales was performed for that test. To explore if specific items in the PDQ-39 subscales were driving the significant improvements, *post-hoc* analyses were made with Related-Samples Wilcoxon Signed Rank Test Summary for each item within the subscales.

#### Qualitative Data Analysis

The qualitative data were thematically analyzed with an inductive approach according to Braun and Clarke (33), which allowed new insights to be derived from the data and ensured that the eventual themes reflected the participants' responses. The Critical Appraisal Skills Program (CASP) checklist for qualitative studies was used as a guidance in reporting the qualitative methodology and results (34). Firstly, the two focus group interviews were transcribed by MD. Data familiarization was conducted by LW, AN, and MD through listening to the audio files and reading the transcripts repeatedly. Early impressions and preliminary codes were formed by LW and discussed with MD and AN. Subsequently, a complete coding of the transcripts was performed by LW using the qualitative data analysis software NVivo 12. Both semantic and latent codes were identified. Themes were generated through a thorough, inclusive and comprehensive coding process and were given preliminary labels. This thematic map was then discussed with MD and AN, after which LW returned to the original data set to compare with the preliminary themes. Additional subthemes and relationships between the themes were discovered, which were then discussed within the group (LW, AN, and MD). This recursive process of persistently reviewing the original data and frequent discussions between LW, AN, and MD ensured rigor in the analysis. Data analysis was completed when LW, AN, and MD agreed upon the themes, subthemes and their relationship, as well as the supporting quotations.

## Results

### Feasibility

In terms of technical and safety issues, 96% gave an average to positive score when considering how the digital dance had worked in general. All participants experienced digital dance as safe and the only side effect mentioned was sore joints, which can be expected after physical exercise. A few participants mentioned technical issues such as unsynchronized sound and limited screen size. Furthermore, a compliance rate of 86% was measured at post-test. Twenty-one (91%) participants completed seven or more dance classes and two participants completed four classes. 83% reported to be highly motivated to attend dance class. Moreover, none of the participants reported worse self-reported physical function, well-being, and cognitive function after 10 weeks of dancing. Lastly, 74% of the participants indicated that they perceived the artistic dimension to digital dance as an important element. In conclusion, results on these feasibility outcome measures suggest that digital dance was feasible in all aspects of feasibility investigated. An overview of the feasibility results can be seen in Table 2.

### Cognitive Complaints, Fatigue, Psychological Health and Quality of Life

There were significant improvements at post-test in PDQ-39 summary index (*p* < 0.001). Subscales Mobility (*p* = 0.016) and Cognition (*p* = 0.005) were significantly improved at post-test. *Post-hoc* analysis for the mobility subscale (10 items) showed that no single item was driving the improvement. For the Cognition subscale (4 items), item 30 (daytime sleepiness) (*p* = 0.008) and item 33 (dreams and hallucination) (*p* = 0.008) showed significant improvements at post-test, but not item 31 (concentration) or item 32 (memory failures). The depression subscale of the HADS questionnaire was significantly improved at post-test (*p* = 0.003) (Table 3). There was no significant change between pre- and post-test for CIS, PRMQ or the two items from the Mental Fatigue Scale.

**Table 3:** Differences on self-reported questionnaires measured at pre- and post-test in 23 persons with PD.

	**Pre-test Mean (SD)**	**Post-test Mean (SD)**	**Change Mean (SD)**	***p*-value**	Cohen's D^*^
**PDQ-39^a^**					
Mobility	35.7 (21.8)	28.6 (23.0)	7.2 (13.2)	0.016	−0.557
ADL	31.3 (19.0)	28.8 (22.2)	2.5 (14.5)	0.410	−0.192
Emotional well-being	31.5 (23.4)	27.5 (20.1)	4.0 (9.9)	0.067	−0.396
Stigma	17.1 (19.9)	13.0 (17.1)	4.1 (13.9)	0.174	−0.278
Social support	11.9 (18.3)	10.5 (19.2)	1.4 (8.2)	0.406	−0.176
Cognition	37.2 (24.1)	29.1 (19.5)	8.2 (12.6)	0.005	−0.624
Communication	26.8 (22.3)	24.3 (20.2)	2.5 (12.4)	0.338	−0.195
Bodily discomfort	42.8 (17.9)	37.3 (18.8)	5.4 (16.8)	0.135	−0.336
Summary index	29.3 (14.7)	24.9 (13.5)	4.4 (4.3)	<0.001	−1.033
**PRMQ total^a^**	35.6 (11.6)	34.0 (10.4)	1.6 (2.2)	0.164	−0.298
**CIS total^a^**	76.0 (23.7)	72.1 (23.1)	4.0 (13.2)	0.164	−0.292
**HADS^a^**					
Depression	4.2 (3.0)	2.9 (2.3)	1.3 (1.8)	0.003	−0.680
Anxiety	5.4 (3.9)	5.4 (3.6)	−0.04 (2.5)	0.934	0.000
					
**Mental exhaustion^b^**	2.3 (0.9)	2.4 (0.8)	−0.17 (0.9)	0.378	0.170
**Mental recovery^b^**	1.7 (0.9	1.8 (0.9)	−0.17 (0.8)	0.305	0.226
					

a *Analyzed with paired sample t-tests*,

b *Analyzed with Wilcoxon Signed Ranked t-test*,

* *Effect size estimates for single group repeated measures design (32)*.

### Focus Groups and Open-Ended Questions

An overview of the themes and sub-categories that were identified in the interview transcripts can be found in Table 4.

**Table 4:** List of themes.

**Name of the theme**
Positivity and thankfulness
A physical awakening
Mental practice makes progress
Music makes me feel good
Home alone
∙ Social contact and community
∙ Individual focus
∙ Importance of the environment

#### Theme: Positivity and Thankfulness

This theme captures the participants' overall experience of digital dance. Participants stated they had enjoyed digital dance and particularly appreciated its practical benefits, such as the ease of access and the lack of preparation needed. This experience of fewer barriers led to an increased ability to participate in digital dance regardless of one's physical or mental state.

“*It's very practical, because it's so easy to sit in front of the computer for a while. There aren't any problems. Otherwise, you might start thinking: I don't have the energy today”* **Participant 4**

Furthermore, an experience of thankfulness was voiced both related to the fact that digital dance was specifically adapted for people with PD and that the participants had received the opportunity to participate. Participants stated that they would recommend digital dance to others with PD, that others should be given the opportunity to participate throughout Sweden, and that it should be provided as a complement to live dance in the future.

Moreover, the importance of digital dance in light of the COVID-19 restrictions was highlighted within this theme. Participants indicated that they had few other physical or social activities and experienced loneliness or a lower mood in general. Digital dance was therefore especially important during such trying times.

”*You can easily feel a bit sad and depressed when you are home alone, especially now when there haven't been any group activities, like exercise groups going on. You're often alone and after dance class you always feel upbeat again.”* **Participant 3**

Answers to the open-ended questions mirrored the theme *Positivity and Thankfulness*. Participants appreciated the practical benefits of digital dance and the ability to participate despite COVID-19. Furthermore, they praised the fact that digital dance was adapted for people with PD and one's individual abilities, e.g., one could participate sitting down.

#### Theme: A Physical Awakening

This theme captures the physical changes that participants experienced, such as better balance, less rigidity and a longer stride length after digital dance. The below passage demonstrates how these physical changes together formed a surreal experience in which one's body became *alive* again through dance.

”*I think that it's amazing because it's as if something magical happens (laughter). You can feel really stiff, that it's a little hard doing the movements. Your body wants to shrivel up and steps become a little trippy. And then you step into this dance and all of a sudden, you're just in it and you're doing everything without any difficulty at all. You know, it's really strange, but fantastic and it's so enjoyable.”* **Participant 3**

Participants described similar physical benefits in the open-ended questions. However, some participants noted that digital dance was not intensive enough and that they had noticed little physical change.

#### Theme: Mental Practice Make Progress

This theme captures how participants experienced digital dance as a cognitively challenging activity. It took effort to learn and remember the different dance steps, which led to an experience of mental tiredness after the dance classes.

”*You sit and do the movements, so you become tired not only physically but also mentally tired and that in itself is actually very beneficial”* **Participant 8**

The above-mentioned comment also demonstrates how the cognitive side of digital dance was valued positively. After the effortful phase of learning the dance steps, the movements became automized and were triggered through hearing the music.

”*Every time you dance, you learn the movements more and more, until eventually you've got them. Now if you would hear the melodies playing on the radio, you'd probably start dancing.”* **Participant 8**

#### Theme: Music Makes Me Feel Good

This theme illustrates the beneficial effect that music has on mood. Positive emotions, such as happiness and optimism, were experienced during and after digital dance. Furthermore, participants indicated feeling more alert and being energized. Such positive feelings were especially linked to the role of music.

“*I always find that I feel better after having danced. Especially when it's to music, some movement to music. That's nearly half the dance, the music that you move to, preferably with a bit of a beat. That's important to me.”* **Participant 2**

Music was even used as a strategy to cope during daily life struggles, which led to an experience of improved mood. This is exemplified by Participant 7 in the following excerpt.

”*When things are hard, when I'm standing in line or something, I can start singing quietly so that no one can hear me, but I hear the melody. Dean Martin and the other old favorites. So, there I've got tools that I can use.”* **Participant 7**

The answers to the open-ended questions are in keeping with this theme. Participants stated that they experienced joy when dancing to music together. They also described that they felt happier after digital dance and appreciated the choice of music.

#### Theme: Home Alone

This theme captures the limitations of digital dance. Within this theme, three sub-themes were identified, i.e., social contact or community, individual focus, and the importance of the environment.

Most importantly, a diminished sense of community or lack of social contact was regarded as a large limitation to digital dance. Participants missed elements such as being able to chat, share experiences about PD and form a social support group. A shortage of time to informally talk to each other and the technology itself interfered in the development of more social contact in the digital platform, as the below discussion between participants 8, 2 and 1 exemplifies.

“*We usually talk a little while, but not so much. If you are logged in, then you can sit and talk to each other when you are waiting for it (dance class) to start. But, it's not that easy. Often, we all talk at the same time, it becomes a bit crazy.”* **Participant 8, 2 and 1**

Within this sub-theme, participants with previous experience of live dance expressed that they missed a sense of community that emerges over time in live dance. This group-process was described as a feeling of security and a deeper connection with each other, which was defined as essential for one to experience dance as an artistic activity.

A second sub-theme to the theme *Home Alone* was the individual focus in digital dance. Participants expressed that it felt peculiar to dance at home in front of their computer or TV. They also stated being aware of passers-by potentially looking in and seeing them. This preoccupation with oneself was closely linked to not being in the same room together with the other dancers.

”*When you are dancing at home by yourself, then you feel a bit odd (general laughter). I mean when you are on the (dance) floor where everyone is in the same room at the same time, then it feels more like you are in a community. In this (digital dance), you become a bit alone and passers-by might look in through the window and wonder what you are doing.”* **Participant 6**

Lastly, a third sub-theme focused on the dance environment. Here, space limitations were experienced as a hinder to move freely at home, i.e., feeling inhibited from making larger movements. Participants with previous experience of live dance stated that they missed the dance floor, as it facilitated self-expression in their dance.

“*There was this big dance floor and “now you may improvise” and it finally felt as if I could take something from within. That was much easier in a room where there are other people than here in the small squares on the screen, because then I look at the person I should be imitating. Instead of taking it from the inside, I try to take it from the outside and it is accentuated by this (digital dance)”* **Participant 7**

As the above excerpt demonstrates, the limitation linked to the digital dance environment negatively affected one's ability to experience digital dance as an art form and an emotional outlet. Participants who had previous experience of live dance also reported such a diminished artistic experience in digital dance due to its individual focus and decreased sense of community.

In the open-ended questions, participants mentioned similar difficulties with digital dance, e.g., not having enough room at home to perform the movements properly, and missing social contact.

## Discussion

The objective of this study was threefold, i.e., to evaluate the feasibility of digital dance for people with PD; to measure change in self-reported quality of life, cognitive complaints, psychological health, as well as fatigue; and to examine the experience of digital dance for people with PD. Our main results showed that digital dance was feasible to complete for people with PD and was experienced as safe. Furthermore, significant improvements in quality of life and depressive symptoms were measured at post-test. Finally, participants described digital dance as a beneficial and enjoyable activity that was especially valued during the COVID-19 pandemic. Nevertheless, they experienced that digital dance missed some important elements of live dance and therefore might benefit from focusing more on these.

Digital home-based healthcare interventions have been regarded as important elements in a person-centered, holistic rehabilitation trajectory for people with PD (35). Such digital interventions may be able to reach out to people with PD and the older population in general, as studies report that they experience both physical and psychological barriers to participate in healthcare interventions, such as exercise (20, 21). However, digital interventions themselves create different obstacles as they rely on technical access and familiarity, making it essential to assess the feasibility of such digital interventions. The feasibility results from the current study demonstrated that digital dance was experienced as safe, that limited technical difficulties arose, and few minor side effects were experienced. These findings are in line with previous studies assessing digital home-based physical exercise interventions for people with PD (23, 24, 36). Nevertheless, it is worth noting that even if the digital dance classes were experienced as safe by all participants, future research should examine the optimal class size for digital dance and take into consideration the dance instructor's ability to actively monitor the participants. Furthermore, a compliance rate of 86% was measured and participants expressed high satisfaction levels as well as benefits related to physical and psychological well-being. Such high utilization and satisfaction with a digital intervention during the COVID-19 pandemic have also been reported by a recent survey study assessing accessibility and engagement with home-based dance programs in people with PD (36), as well as in patients with cancer who participated in digital dance and other mind/body interventions (37).

Results from the pre-post questionnaires showed a significant improvement in quality of life and depressive symptoms, which is in keeping with previous work (14, 15). As there is no control group, these results should be interpreted with caution. Nevertheless, it is interesting to note that the improvements are not general across domains, but selective and with medium to large effects sizes. In particular, significant improvements in the subdimension of mobility and cognition (PDQ-39) were seen at post-test. The measured change in self-reported mobility is in line with the findings that dance has a positive effect on functional mobility (9, 10, 12, 13). Concerning the cognition subscale of the PDQ-39, *post-hoc* analyses displayed that it was especially the items linked to daytime sleepiness and dreams/hallucinations that drove this improvement. Findings from the focus groups support this improvement in daytime sleepiness, as participants indicated they felt energized and alert after digital dance. In addition, previous studies have reported the beneficial effect of physical exercise on sleep in people with PD (38). The fact that the cognition subscale items on concentration and memory failures did not drive the measured change is in agreement with the lack of change seen in the PRMQ.

The qualitative results extended the above-mentioned findings through examining the experience of digital dance for people with PD. Firstly, participants focused on the benefits of digital dance, which were captured in several themes that described participants' positive experience of digital dance, its importance during the COVID-19 pandemic as well as its benefits on both physical and psychological health. Participants stressed that they appreciated the ease of participation in digital dance. They experienced fewer psychological or physical barriers to participate in digital dance, which has also been reported by Bek et al. (36). Furtheremore, the importance of music and its positive effect on mood was also raised by the participants. It is of interest that several of these themes have been observed in studies assessing live dance for people with PD (e.g., the physical changes and the importance of music) (18, 19), suggesting that digital and live dance may share similar qualities.

However, participants noted several limitations to digital dance, i.e., lack of social contact, focus on the individual, and space issues. Firstly, the importance of social contact and becoming a group has been noted in numerous studies on dance for people with PD (18, 36, 39–41). Furthermore, the degree to which a dancer takes on an internal or external focus within their dance has shown to influence their ability to perform the movements (42). The increased individual focus that participants reported in digital dance may have prevented them from fully embracing the dance. Finally, context played an important role in the participants' experience of digital dance, as they expressed a strong desire to return to the dancefloor. Such context-related limitations with digital dance have been reported in previous work (36). Participants who had previously participated in live dance indicated that these three limitations to digital dance had a negative effect on one's ability to experience dance as an artistic activity.

The following limitations to the study can be noted. Firstly, the single-group pre-post design makes it impossible to rule out a placebo effect. Nevertheless, the measured changes in quality of life correspond to previous findings from controlled trials on dance in people with PD (43, 44) and have been perceived as meaningful improvements by people with PD (45). Furthermore, the study sample is small and consists of a select sample of highly educated people (83% with a university degree) with high levels of motivation to participate in digital dance, leading to limitations in generalizability. Moreover, the study relied on self-reported PD diagnosis and was not confirmed by medical records. Nor did the study collect information on the disease stage of the participants, which is essential information when implementing digital dance as a health intervention. Nevertheless, the current population had a disease duration ranging from one to 26 years, which alludes to digital dance being feasible and experienced as safe for a wide range of people with PD. In addition, future studies are encouraged to use both objective and subjective measures to better understand the changes seen after digital dance interventions. Lastly, the COVID-19 restrictions to the participants' daily life could have formed both an increased incentive to participate and a specific context in which the impact of digital dance was especially beneficial, potentially leading to an overestimation of the participant's positive experience. However, beyond COVID-19, many people with PD experience a decreased ability to participate in society (46). Hence, examining the effects of digital dance through randomized controlled trials in a larger population of people with PD remains an important task even after the COVID-19 pandemic.

In conclusion, this study shows that digital dance is a feasible activity for people with PD that was experienced as safe. Moreover, our findings demonstrate that dance is more than just physical exercise. Quantitative findings showed improvements in self-reported mobility, quality of life and depressive symptoms, while participants also voiced positive effects on physical abilities, cognition and mood in the focus groups. Such findings are in line with previous studies on dance (9, 14, 18, 19). However, participants drew attention toward several limitations in digital dance. This study encourages future digital dance classes to focus more on the environment in which one dances digitally, to increase social contact between the participants aiming to develop a sense of community, as well as trying to decrease the individual focus that can occur in digital dance.

## Data Availability Statement

The raw data supporting the conclusions of this article will be made available by the authors, without undue reservation.

## Ethics Statement

This study was reviewed and approved by the Swedish Ethical Review Authority (Dnr 2020-00544). The research was performed in compliance with the Helsinki Declaration. Informed consent was given by all participants before answering the online questionnaire.

## Author Contributions

MD and AN were responsible for the study design and data collection. LW performed the qualitative analysis and wrote the manuscript with support from AN and MD. MD performed the statistical analyses. LW, MD, and AN interpreted the data. MD, AN, and ÅÅ were responsible for the recruitment. ÅE and ÅÅ were responsible for the dance classes. All authors have revised the manuscript critically for important intellectual content and approved it for publication.

## Funding

This work was supported by the Center for Arts and Health, Region Stockholm, Sweden (Grant Number 136/2019) and by the Swedish Research Council (2017-02371).

## Conflict of Interest

The authors declare that the research was conducted in the absence of any commercial or financial relationships that could be construed as a potential conflict of interest.

## Publisher's Note

All claims expressed in this article are solely those of the authors and do not necessarily represent those of their affiliated organizations, or those of the publisher, the editors and the reviewers. Any product that may be evaluated in this article, or claim that may be made by its manufacturer, is not guaranteed or endorsed by the publisher.

## Abbreviations

PD: Parkinson's Disease
DRT: Dopamine Replacement Therapy
DfPD: Dance for Parkinson's Disease
HADS: Hospital Anxiety and Depression Scale
PRMQ: Prospective and Retrospective Memory Questionnaire
PDQ-39: Parkinson's Disease Questionnaire
CIS: Checklist Individual Strength.
